# Preparation and Evaluation of Core–Shell Magnetic Molecularly Imprinted Polymers for Solid-Phase Extraction and Determination of Sterigmatocystin in Food

**DOI:** 10.3390/polym9100546

**Published:** 2017-10-23

**Authors:** Jing-Min Liu, Shu-Yuan Wei, Hui-Lin Liu, Guo-Zhen Fang, Shuo Wang

**Affiliations:** 1 Beijing Advanced Innovation Center for Food Nutrition and Human Health, Beijing Technology & Business University (BTBU), Beijing 100048, China; liujingmin@nankai.edu.cn (J.-M.L.); liuhuilin1290@163.com (H.-L.L.); 2 Research Center of Food Science and Human Health, School of Medicine, Nankai University, Tianjin 300071, China; 3 Key Laboratory of Food Nutrition and Safety, Ministry of Education, Tianjin University of Science and Technology, Tianjin 300457, China; nkbluer@163.com (S.-Y.W.); fangguozhen@tust.edu.cn (G.-Z.F.)

**Keywords:** sterigmatocystin, molecularly imprinted polymers, magnetic nanoparticles, solid-phase extraction, HPLC

## Abstract

Magnetic molecularly imprinted polymers (MMIPs), combination of outstanding magnetism with specific selective binding capability for target molecules, have proven to be attractive in separation science and bio-applications. Herein, we proposed the core–shell magnetic molecularly imprinted polymers for food analysis, employing the Fe_3_O_4_ particles prepared by co-precipitation protocol as the magnetic core and MMIP film onto the silica layer as the recognition and adsorption of target analytes. The obtained MMIPs materials have been fully characterized by scanning electron microscope (SEM), Fourier transform infrared spectrometer (FT-IR), vibrating sample magnetometer (VSM), and re-binding experiments. Under the optimal conditions, the fabricated Fe_3_O_4_@MIPs demonstrated fast adsorption equilibrium, a highly improved imprinting capacity, and excellent specificity to target sterigmatocystin (ST), which have been successfully applied as highly efficient solid-phase extraction materials followed by high-performance liquid chromatography (HPLC) analysis. The MMIP-based solid phase extraction (SPE) method gave linear response in the range of 0.05–5.0 mg·L^−1^ with a detection limit of 9.1 µg·L^−1^. Finally, the proposed method was used for the selective isolation and enrichment of ST in food samples with recoveries in the range 80.6–88.7% and the relative standard deviation (RSD) <5.6%.

## 1. Introduction

Sterigmatocystin (ST), a carcinogenic mycotoxin, is a secondary metabolite produced mainly by *Aspergillus versicolor*, *Aspergillus flavus*, *Aspergillus nidulans*, fine wrinkles and other fungi *Aspergillus* [1,2,3]. With a basic structure composed of two furan rings with oxygen hetero anthraquinone, ST possess a similar structure to Aflatoxin B1 and its toxicity is second only to Aflatoxin that can induce liver cancer, lung cancer and other cancers [4,5]. Moreover, ST spreads widely in nature, and could contaminate most food and forage, especially wheat, corn, peanuts and forage, raising great concerns of public society [6,7]. For the quantification of ST in food samples, usually with complex matrix, liquid–liquid extraction or solid phase extraction coupled with high-performance liquid chromatography-mass spectrometry (HPLC-MS) has come to be an effective solution, of which the analytical performance highly depends on the adsorption materials [1].

Molecularly imprinted polymers (MIPs) have attracted much attention in the past decades for their high affinity and selectivity [8,9,10]. Molecular imprinting is a well-known method to create recognition sites structurally complementary to the target molecules in a synthetic polymer [11,12,13]. Generally, MIPs are fabricated via polymerization in the presence of template molecules followed by removal of the template. After the templates are wiped out, the remaining cavities are left behind which have complementary shape, size, and orientation of functionalities [14,15,16]. MIPs obtained with the memory of size, shape, and functional groups of the target molecules possess notable advantages, such as outstanding specificity, improved stability, cost-effective, ease of fabrication, and reversible adsorption/release, qualifying MIPs as ideal SPE materials [17,18].

In recent years, with the involvement of nano/micro-structures as functional core, such as magnetic Fe_3_O_4_ nanoparticles [19], quantum dots [20,21], carbon nanomaterials [22], and upconversion nanoparticles [23,24], surface imprinting technique appeared as the advantageous protocol for fabrication of advanced MIPs materials with diverse functionalities. Therein, magnetic molecularly imprinted polymers (MMIPs), prepared via fabrication of the MIPs on the surface of a magnetic substrate, combined the outstanding magnetism with specific selective binding capability for target molecules, favoring rapid and easy removal of magnetic polymers from sample matrix by applying a magnetic field without the need for tedious filtration or centrifugation [25,26,27,28]. Compared with conventional MIPs, MMIPs demonstrate several superior features involving fast and effective binding to target analytes, magnetically susceptible characteristic, shorter pretreatment time, and reversible and controllable flocculation [29,30]. The magnetic separation process can be performed directly in crude samples containing suspended solid or other biological particles in a rapid and simple way, thus greatly saving time and cost. Therefore, a combination of magnetic separation and molecular imprinting would generate a powerful analytical method with simplicity, flexibility, and selectivity, especially as SPE adsorbents for complex sample analysis [28].

Herein, we proposed the core–shell MMIPs for food analysis, employing the Fe_3_O_4_ particles prepared by co-precipitation protocol as the magnetic core and MIP film onto the silica layer as the recognition and adsorption of target analytes. First, Fe_3_O_4_ particles were prepared by a solvothermal reduction method. Then, silica shell was deposited by adding tetraethyl ortosilicate (TEOS) and ammonia. Subsequently, the vinyl groups were grated onto silica-modified Fe_3_O_4_ surface by 3-methacryloyloxypropyltrimethoxysilane (MPS). The MIPs were coated on the Fe_3_O_4_@SiO_2_ by the copolymerization of vinyl end groups with functional monomer, acrylamide, cross-linking agent, ethylene glycol dimethacrylate (EGDMA), the initiator, azobisisobutyronitrile (AIBN) and dummy template molecule, 1,8-dihydroxyanthraquinone (DT). The obtained MMIPs materials have been fully characterized by scanning electron microscope (SEM), Fourier transform infrared spectrometer (FT-IR), vibrating sample magnetometer (VSM), and re-binding experiments. Under the optimal conditions, the fabricated Fe_3_O_4_@MIPs demonstrated fast adsorption equilibrium, a highly improved imprinting capacity, and excellent specificity to target ST, which have been successfully applied as highly efficient solid-phase extraction materials followed by HPLC analysis. The MMIP-based SPE method gave linear response in the range of 0.05–5.0 mg·L^−1^ with a detection limit of 9.1 µg·L^−1^.

## 2. Materials and Methods

### 2.1. Chemicals and Materials

All the reagents used were of analytical or HPLC grade. Tetraethyl ortosilicate (TEOS) was procured from Wuhan University Silicone New Material Co., Ltd. (Wuhan, China). Acrylamide (AM) was obtained from Tianjin Chemical Reagent Research Institute (Tianjin, China). Sterigmatocystin (99%), DT, zearalenone (ZEN), aflatoxin B1 (AFB1), aflatoxin B2 (AFB2), aflatoxin M1 (AFM1), microcystin-leucine-arginine (MC-LR), ochratoxin A (OTA), vomitoxin (DON), and EGDMA (98%) were obtained from Sigma-Aldrich (St. Louis, MO, USA). 2,2-azobisisobutyronitrile (AIBN; 99%) was purchased from Tianjin Kermel Chemical Reagents Co. Ltd. (Tianjin, China). Acetonitrile, methanol, ethanol, *n*-hexane, acetone, and glacial acetic acid were obtained from Sinopharm Group Co. Ltd. (Tianjin, China). Iron (II) dichloride and iron (III) chloride were obtained from Chemicals Co. Ltd. (Tianjin, China). Highly purified water was obtained from a Pro Water System (Millipore Co. Ltd., Billerica, MA, USA). The food samples were randomly obtained from some local supermarkets (Tianjin, China). The samples of wheat and rice millet were purchased from a local supermarket (Tianjin, China). The ST-free samples were detected by HPLC. Caution is necessary when operating with toxins, wear the necessary personal protective equipment (PPE) including gloves and protective facial mask.

### 2.2. Instrumentation

Ultraviolet–visible (UV–vis) spectra over 200–800 nm was recorded on an Evolution 300 spectrophotometer (Thermo Fisher Scientific, Waltham, MA, USA). Scanning electron microscopy (SU 1510, Hitachi, Tokyo, Japan) was used to observe the shape, size and surface morphologies of Fe_3_O_4_, Fe_3_O_4_@SiO_2_, Fe_3_O_4_@MIPs and Fe_3_O_4_@NIPs (non-imprinted polymers). Transmission electron microscope (TEM) images were obtained on a 2010 FEF microscope (JEOL, Tokyo, Japan). The infrared spectra were observed using a Tensor 27 FT-IR spectrophotometer (Bruker Company, Berlin, Germany). The magnetic intensity was evaluated using a 7410 VSM (Lake Shore Company, MA, USA). The analysis of HPLC was performed using HPLC (Shimadzu, Tokyo, Japan) with a variable wavelength UV–visible detector. A ZORBAX Eclipse XDB-C_18_ (5 μm, 250 mm × 4.6 mm, Shimadzu, Tokyo, Japan) analytical column was used for the separation of analytes. The mobile phase was methanol/H_2_O (4/1, *v*/*v*), and the flow rate was 0.7 mL·min^−1^ at 35 °C. The injection volume was 20 μL, and the wavelength of the UV detector was proceeding at 246 nm.

### 2.3. Synthesis of Silica Coated Fe_3_O_4_ Particles

Referring to the reported solvothermal method [31], the FeCl_3_·6H_2_O (1.35 g, 5 mmol) was dissolved in ethylene glycol (EG, 40 mL), with continued stirring to form a clear solution, followed by the addition of NaAc (3.6 g) and polyethylene glycol (1.0 g). The mixture solution was stirred vigorously for another 30 min, and sealed in a teflon lined stainless-steel autoclave. The autoclave was heated to 200 °C and maintained for 8 h, then cooled to room temperature after complete reaction. The black products were washed three times using ethanol with the assistance of external magnet isolation. The obtained magnetic Fe_3_O_4_ particles product was dried at 60 °C for 6 h (0.26 g with a yield of 68.4%).

Thirty mg of Fe_3_O_4_ particles were dispersed in ethanol (50 mL), and 0.4 mL of TEOS was added, followed by cautious addition of 2 mL of ammonia water (20%) dropwise. The reaction was allowed to proceed under continuous stirring for 8 h at room temperature, and the resultant product was repeatedly washed four times using ethanol by external magnets, dried in a vacuum oven at 60 °C for 10 h (38.4 mg with a yield of 86.7%).

One hundred mg of thin silicon coated Fe_3_O_4_ particles were dispersed in isopropanol (18 mL), followed by addition of 0.5 mL of aqueous ammonia solution (30%) under continued stirring. Then, excessive γ-methacryloxypropyl triethoxysilane (γ-MAPs) was added drop by drop, and the mixture was allowed to react for 24 h at room temperature under continuous stirring. The resultant product was collected by an external magnetic field and rinsed with ethanol four times thoroughly, dried in the vacuum (98.2 mg with a yield of 98.2%).

### 2.4. Preparation of Magnetic Core–Shell Fe_3_O_4_@MIPs and Fe_3_O_4_@NIPs Particles

The MIPs were prepared by a precipitation polymerization approach. First, DT (120 mg) and acrylamide (AM) (142.2 mg) was mixed with 30 mL of acetonitrile, with continuous stirring until completely dissolved, followed by addition of 100 mg of Fe_3_O_4_@SiO_2_-MAPs. The mixed solution was kept under ultrasonic treatment (300 W, SB-5200D, SCIENTZ, Ningbo, China) for 15 min followed with forming template-monomer complex at 25 °C for 3 h. EGDMA (1.981 g) and AIBN (60 mg) were added and then mixed the solution adequately with a 15-min ultrasonic treatment. The mixed solution was transferred to a 250 mL three-necked flask followed by the addition of 70 mL of acetonitrile. Finally, nitrogen was purged in to remove oxygen and reaction proceeded by thermal polymerization at 60 °C for 24 h. The products were thoroughly grinded, filtrated through a 200-mesh sieve, and treated with 200 mL of acetone and acetonitrile (9:1, *v*/*v*) to remove the template. The washing efficiency was evaluated via UV–vis measurement (429 nm) of DT amount in supernatant solutions. Procedure of preparation of non-imprinted Fe_3_O_4_@NIPs was the same to that of Fe_3_O_4_@MIPs in the absence of the template DT. The obtained polymer products (MIP ~680 mg, NIP ~710 mg) was thoroughly washed by ethanol three times and dried under vacuum.

### 2.5. Determination of Sterigmatocystin via Magnetic Fe_3_O_4_@MIPs

To investigate the recognition properties of Fe_3_O_4_@MIPs and Fe_3_O_4_@NIPs, the adsorption experiments were performed. Every test was performed thrice in parallel. Blank tests were performed by incubating MIP or NIP with water and measuring by HPLC to ensure there was no interferent existing in the polymers.

In the adsorption kinetics experiment, 5 mg of Fe_3_O_4_@MIPs or Fe_3_O_4_@NIPs were added to 3 mL of ST solution (20 mg·L^−1^), and incubated at regular time intervals from 10 min to 300 min at room temperature. After separating the supernatants and polymers using an external magnetic field, the concentration of ST in the supernatants was measured by a UV–visible spectrophotometer (327 nm). The amount of ST bound to the Fe_3_O_4_@MIPs or Fe_3_O_4_@NIPs was figured out by the formula.

In the isothermal binding experiment, 5 mg of Fe_3_O_4_@MIPs or Fe_3_O_4_@NIPs were added to 3 mL methanol solution of ST of various concentrations from 2 to 50 mg·L^−1^ and the mixture was incubated for 10 min at room temperature respectively. After incubation, the supernatants and polymers were separated by an external magnet, and the remaining ST in the supernatants was determined by UV–visible spectroscopy at 327 nm.

In the selectivity experiments, AFB1 was selected as the structural analog. The Fe_3_O_4_@MIPs or Fe_3_O_4_@NIPs (25 mg) was placed in methanol solution of ST or AFB1 (20 mg·L^−1^, 10 mL). After incubating for 4 h at room temperature, the supernatant and polymers were separated using an external magnetic field and the concentration of ST and its analog in the supernatant was determined by HPLC–UV. Moreover, to further verify the competitive recognition ability, Fe_3_O_4_@MIPs (20 mg) was mixed with 10 mL methanol solution of ST and AFB1 (20 mg·L^−1^ each). The extraction and determination procedures were then performed as described earlier for the static adsorption experiments. The same procedure was performed for the Fe_3_O_4_@NIPs.

### 2.6. Adsorption Isotherms

The Langmuir and Freundlich isotherm models were employed to evaluate the adsorption process. The widely used Langmuir equation, which was valid for monolayer sorption on a surface with a finite number of identical sites, is given by

(1) $$\frac{C_{e}}{q_{e}}=\frac{C_{e}}{Q_{m}}+\frac{1}{Q_{m}K_{l}}$$

where *Q_m_* (mg·g^−1^) is the maximum adsorption capacity of adsorbent at monolayer, and *K_l_* is the Langmuir constants.

The essential characteristics of the Langmuir isotherm can also be expressed in terms of

(2) $$R_{l}=\frac{1}{1+K_{l}C_{0}}$$

where *R_l_* is a dimensionless constant of separation factor or equilibrium parameter, which indicates the shape of adsorption isotherm.

The widely used empirical Freundlich equation, based on sorption on a heterogeneous surface, is given as:

(3) $$q_{e}=K_{F}{C_{e}}^{1/n}$$

where *K_F_* and *n* are Freundlich constants indicating adsorption capacity and intensity, respectively, which can be calculated from linear plot of ln *q_e_* against ln *C_e_*.

### 2.7. Adsorption Kinetics Investigations

To investigate the mechanism of sorption and potential rate controlling steps, the pseudo-first-order, pseudo-second-order, intra-particle diffusion and Elovich model have been used to test the experimental data. The rate constants for four models have been determined and the correlation coefficients have been calculated in order to assess which model provides the best fit of the predicted data with the experimental results.

The pseudo-first-order kinetic model known as the Lagergern equation:

(4) $$\frac{dq}{dt}=K_{1}\left(q_{e}-q_{t}\right)$$

where *q_t_* and *q_e_* are the amounts of ion adsorbed at time *t* and at equilibrium (mg·g^−1^), respectively, and *K*_1_ is the rate constant of pseudo-first-order adsorption process (min^−1^). After integration and applying boundary conditions, for *t* = 0, *q* = 0, the integrated form of equation becomes:

(5) $$\ln\left(q_{e}-q_{t}\right)=\ln q_{e}-K_{1}t$$

The pseudo-second-order equation based on adsorption equilibrium capacity can be expressed as:

(6) $$\frac{dq}{dt}=K_{2}{\left(q_{e}-q_{t}\right)}^{2}$$

where *K*_2_ is the rate constant of pseudo-second-order sorption (g·mg^−1^·min^−1^). For *t* = 0, *q* = 0, it was given as:

(7) $$\frac{t}{q_{t}}=\frac{1}{K_{2}{q_{e}}^{2}}+\frac{t}{q_{e}}$$

The Elovich equation was also be applied to analyze the adsorption data. Its linear form was given as:

(8) $$q_{t}=\frac{1}{\beta }\ln\left(\alpha \beta \right)+\frac{1}{\beta }\ln\left(t\right)$$

where *α* is the initial sorption rate constant (mg·g^−1^·min^−1^), and the parameter *β* is related to the extent of surface coverage and activation energy for chemisorption (g·mg^−1^). *α*, *β* can be obtained from the slope and intercept of the plot of *q_t_* versus ln *t*.

The intra-particle diffusion model was also tested. The initial rate of intra-particle diffusion is as follows:

(9) $$q_{t}=k_{int}t^{1/2}+C$$

where *k_int_* is the intra-particle diffusion rate constant (mg·g^−1^·min^−1/2^), and *C* is the intercept.

### 2.8. Real Sample Analysis

All food samples were free of ST, and the spiking concentrations were 50 μg·kg^−1^, 100 μg·kg^−1^, and 200 μg·kg^−1^. The food sample (1.0 g) was accurately weighed in a 25-mL conical flask with a stopper, and then different amounts of ST standard solutions were added (5 mg·L^−1^, 50 μL, 100 μL, and 200 μL, dissolved in methanol). After thoroughly incorporated, the mixture stands overnight. Ten milliliters of *n*-hexane were added, and the mixture was treated by ultrasonic for 10 min. Exactly 3.0 mL of the supernatant solution was added to another 25-mL conical flask. Typically, 15.0 mg of Fe_3_O_4_@MIPs were added and shaken at 120 rpm for 50 min at room temperature on a shaker (MS 3 digital, IKA, Berlin, Germany). The supernatant and polymers were separated using an external magnetic field. After removing the supernatant solution, the Fe_3_O_4_@MIPs was washed with 1 mL of *n*-hexane/ether (4:1, *v*/*v*) to eliminate the co-extracted impurities. Then, the ST was eluted from the Fe_3_O_4_@MIPs with 3 × 1.0 mL of chloroform, and the elutes were combined together and evaporated to almost dryness under a stream of nitrogen. The residue was re-dissolved by 1.0 mL of chromatographic pure methanol, and filtered through a 0.45 μm organic membrane, and finally detected by HPLC.

## 3. Results and Discussion

### 3.1. Preparation and Characterization of Imprinted Magnetic Nanoparticles

**Figure 1:**
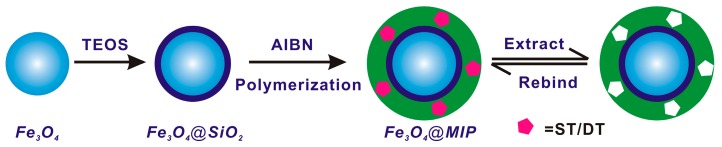
Schematic illustration of Fe_3_O_4_-involved magnetic molecularly imprinted polymers for sensitive and specific recognition of sterigmatocystin. TEOS: tetraethyl ortosilicate; AIBN: 2,2-azobisisobutyronitrile; MIP: molecularly imprinted polymers; ST: sterigmatocystin; DT: 1,8-dihydroxyanthraquinone.

When constructing the MIP materials, a dummy template molecule is often employed as an alternative template for imprinting instead of the target analyte, in the case that the target analyte is highly toxic, expensive or unavailable [15,32]. The dummy template should possess the similar structure and physicochemical property to the target molecules and scarcely present in the related sample matrix to avoid possible interference. In the previously-reported work, DT was a widely-used dummy template molecule for imprinting of ST, due to the high price and toxicity of ST [22,24]. Therefore, the magnetic molecularly imprinted polymers for recognizing ST specifically were synthesized using DT as the dummy template molecule, which has similar structure with ST and hardly presents in the grain samples. The preparation procedure of Fe_3_O_4_@MIPs by the precipitation polymerization is shown in Figure 1. Super paramagnetic Fe_3_O_4_ nanoparticles were synthesized by the solvothermal method to provide a good magnetic core, supporting the MIPs material magnetic response. The surface of the Fe_3_O_4_ core was encapsulated with SiO_2_ shell by TEOS to avoid the oxidation and provide silanol groups at the surface, which make them biocompatible and easily modified with various functional groups. Furthermore, silica shell could not only shield the magnetic dipolar attraction between magnetic particles, in favor of the dispersion of magnetic particles in solvent, but also protect Fe_3_O_4_ from dissolving in an acidic environment.

To introduce vinyl groups, the hydroxyl groups on surface of Fe_3_O_4_@SiO_2_ were further reacted with MPS, which subsequently reacted in the synthesis of MIPs or NIPs. In addition, the Fe_3_O_4_@SiO_2_ particles had a small diameter with an extremely high surface area-to-volume ratio, so that the MIPs formed easily at the surface of the magnetic particles. The MIPs shells were coated on the surface of Fe_3_O_4_@SiO_2_ by the copolymerization of functional monomer (AM), cross-linking agent (EGDMA), initiator (AIBN) and template molecule (DT). After removal of the templates, the Fe_3_O_4_@MIPs particles were obtained. Overall, the Fe_3_O_4_@MIPs particles could recognize and adsorb the targets effectively and were also easily collected using an external magnetic field. Meanwhile, Fe_3_O_4_@NIPs were also prepared with the same procedure, but without the addition of template DT.

**Figure 2:**
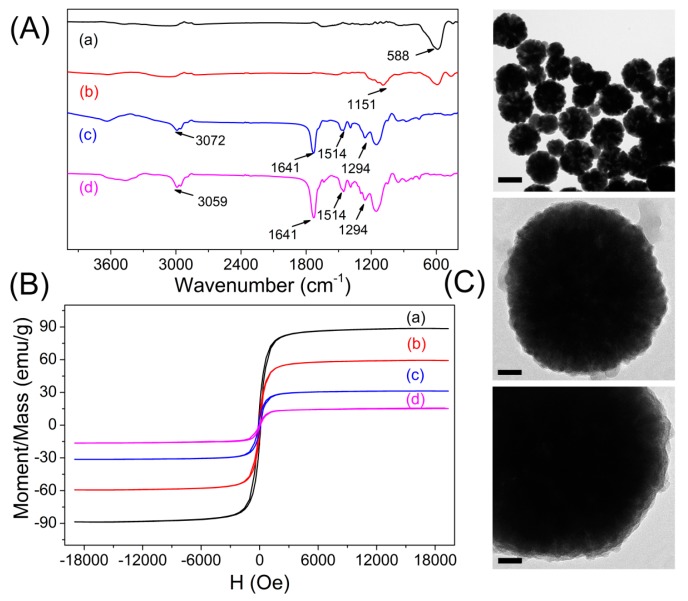
(**A**) Fourier transform infrared spectrometer analysis of: Fe_3_O_4_ (a); Fe_3_O_4_@SiO_2_ (b); Fe_3_O_4_@MIPs (molecularly imprinted polymers) (c); and Fe_3_O_4_@NIPs (non-imprinted polymers) (d). (**B**) Magnetization curves of: Fe_3_O_4_ (a); Fe_3_O_4_@SiO_2_ (b); vinyl-modified Fe_3_O_4_ (c); and Fe_3_O_4_@MIPs (d). (**C**) Typical high-resolution transmission electron microscopy (HRTEM) photographs of the Fe_3_O_4_ (top, scale bars = 300 nm), and Fe_3_O_4_@SiO_2_ (middle and bottom, scale bars = 50 nm). H: magnetic field.

The FT-IR spectroscopy of Fe_3_O_4_, Fe_3_O_4_@SiO_2_, Fe_3_O_4_@MIPs and Fe_3_O_4_@NIPs is shown in Figure 2A. The Fe–O stretching vibration can be observed at 588·cm*^−^*^1^. Compared with the absorption bands of pure Fe_3_O_4_, the characteristic absorption peaks of Si–O–Si at 1151·cm*^−^*^1^ and O–H group at 1641·cm*^−^*^1^ confirmed the formation of silica on the surface of Fe_3_O_4_ after the modification with TEOS and MAPs. Peaks of C=O stretching vibration at 1641 cm*^−^*^1^ and C–H stretching vibration of the methyl group at 3072 cm*^−^*^1^ indicated that the AM-EGDMA layer was successfully formed on the surface of Fe_3_O_4_@SiO_2_. In addition, Fe_3_O_4_@MIPs and Fe_3_O_4_@NIPs showed almost the same characteristic absorption bands, revealing the complete removal of templates. These results proved the successful preparation of MIPs and NIPs on the surface of Fe_3_O_4_@SiO_2_.

Magnetic property is crucial to the magnetic particles for their application in fast separation. VSM was employed to study the magnetic properties of the synthesized magnetic particles, and the magnetic hysteresis loop of the dried samples at room temperature is illustrated in Figure 2B. It is obvious that there is no hysteresis, both remanence and coercivity are zero, suggesting that the samples are superparamagnetism. The saturation magnetization values obtained at room temperature were 88.73 emu·g*^−^*^1^, 59.45 emu·g*^−^*^1^, 31.36 emu·g*^−^*^1^, and 15.17 emu·g*^−^*^1^ for Fe_3_O_4_, Fe_3_O_4_@SiO_2_, vinyl-modified Fe_3_O_4_@SiO_2_ and Fe_3_O_4_@MIPs, respectively. The theoretical value of saturation magnetization for bulk magnetite is reported to be 92 emu·g^−1^. The decrease in magnetization value can be attributed to the small particle surface effect such as magnetically inactive layer containing spins that are not collinear with the magnetic field. The saturation magnetization of Fe_3_O_4_@MIPs was reduced to 15.17 emu·g^−1^ in comparison with the pure Fe_3_O_4_, but remained strongly magnetic at room temperature and qualified as effective magnetic separation carriers.

**Figure 3:**
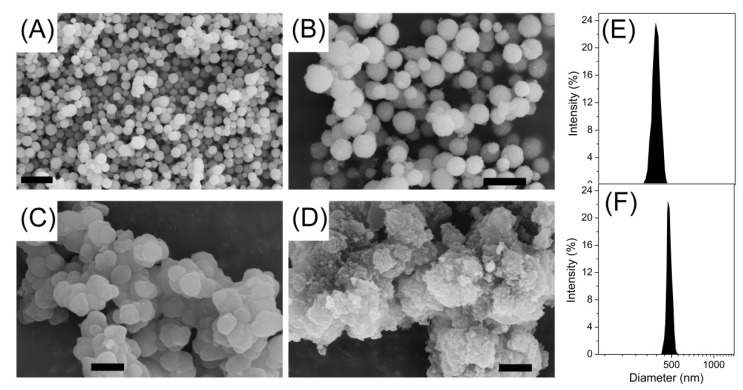
The typical scanning electron microscopy (SEM) photographs of the as-prepared particles: Fe_3_O_4_ (**A**); Fe_3_O_4_@SiO_2_ (**B**); Fe_3_O_4_@MIPs (**C**) and Fe_3_O_4_@NIPs (**D**); size distribution of the Fe_3_O_4_ (**E**); and Fe_3_O_4_@SiO_2_ (**F**). The scale bars all represent 1 µm.

TEM, SEM, and dynamic light scattering (DLS) characterizations revealed that the as-synthesized Fe_3_O_4_ particles possessed an average diameter of ~370 nm. After the modification with TEOS, a thin silicon layer coated on the surface of Fe_3_O_4_ could be distinguished, with the size increased to ~480 nm, which also confirmed the successful preparation of the core–shell structure Fe_3_O_4_@SiO_2_. In comparison, the Fe_3_O_4_@MIPs, with an average diameter of ~830 nm, structures seemed more rough and dense than the Fe_3_O_4_@NIPs (average diameter of ~1.23 µm), indicating the template molecule showed obvious influence over the surface topography. Consequently, this uniform structure of MIP materials would facilitate the mass transport between solution and the shell surface of Fe_3_O_4_@MIPs as SPE adsorbents (Figure 2C and Figure 3).

**Figure 4:**
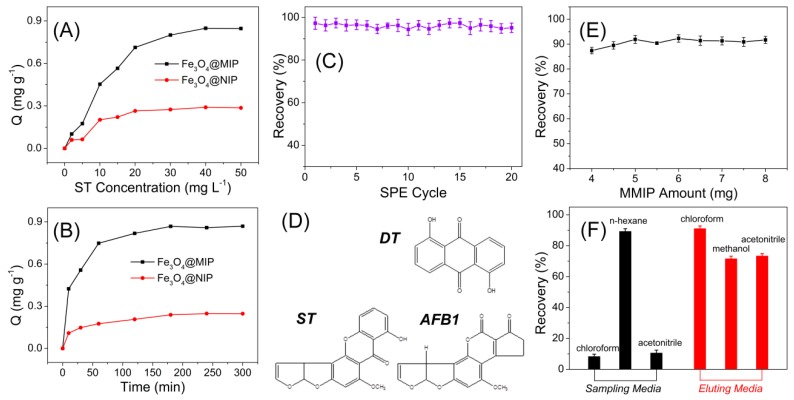
(**A**) Adsorption isotherms of Fe_3_O_4_@MIPs and Fe_3_O_4_@NIPs to ST; (**B**) kinetic uptake plot of Fe_3_O_4_@MIPs and Fe_3_O_4_@NIPs to ST; (**C**) evaluation of reusability of the mean squared prediction error (MSPE) column for ST analysis, with experimental conditions: 5 mg of polymers, 3 mL of ST methanol solution (20 mg·L^−1^ for kinetic uptake; 2 to 50 mg·L^−1^ for adsorption isotherms), 25 °C; (**D**) the structures of DT, ST and aflatoxin B1 (AFB1); (**E**) optimization of magnetic molecularly imprinted polymers (MMIP) amount for solid phase extraction (SPE); and (**F**) optimization of sampling media and eluting media for SPE.

Experimental conditions, including MMIPs amount and organic media used for SPE assay, have been fully optimized. Results in Figure 4 indicated the MMIPs amount showed insignificant influence over the recovery, thus chose the relative small amount of 5 mg for all assays. When using *n*-hexane as the sampling media and chloroform as the eluting media, best SPE performance was achieved. It was also found that pH showed little influence on the adsorption, probably because there was limited electrostatic interaction in the adsorption process that carried out all in non-aqueous solvents.

### 3.2. MIPs Recognition of Sterigmatocystin

The binding isotherms of ST onto Fe_3_O_4_@MIPs and Fe_3_O_4_@NIPs were determined in the concentration range of 0–50 mg·L*^−^*^1^, and the results are shown in Figure 4A. Molecular recognition of Fe_3_O_4_@MIPs and Fe_3_O_4_@NIPs particles increased rapidly with increasing initial concentration, and became relatively flat and reached its saturation at high ST concentration. The amount of ST bound to the Fe_3_O_4_@MIPs was significantly higher than that of the Fe_3_O_4_@NIPs at the same initial concentration. The recognition ability of Fe_3_O_4_@MIPs particles towards ST was investigated by adsorption kinetics. The adsorption kinetics of 20 mg·L*^−^*^1^ ST solution to Fe_3_O_4_@MIPs and Fe_3_O_4_@NIPs are shown in Figure 4B. The adsorption capacity increased with time, and the Fe_3_O_4_@MIPs showed a fast adsorption rate. The adsorption capacity increased rapidly in the first 30 min and almost reached equilibrium after 2 h. Most of the recognition sites of the imprinted polymers are on the surface of the imprinted magnetic particles, facilitating high adsorption efficiency.

The adsorption process was interpreted both with Langmuir and Freundlich isotherm models. Comparison of the calculated data from Langmuir and Freundlich isotherm models indicated the obtained data is better fitted with Langmuir model than with Freundlich model, revealing the adsorption was more similar to a monolayer adsorption process rather than a multiple process. The Langmuir constant *R_l_* was in the range of 0–1, which indicated favorable adsorption of MMIP to the analytes (Table 1). The models of the pseudo-first-order, pseudo-second-order, intra-particle diffusion and Elovich model were employed to evaluate the kinetic mechanism. It was noticed that pseudo-second-order equation provided the better *R*^2^ and agreement between calculated *q_e_* values and the experimental *q_e_* (*exp*) value (0.95), whereas the pseudo-first-order, Elvoich and intra-particle diffusion equations did not give a good fit to the experimental data for the adsorption of ST (Table 1).

The regeneration ability and the stability of Fe_3_O_4_@MIPs materials were evaluated by spacing and non-spacing adsorption and elution cycles, and the SPE efficiency was assessed by observing the changes of the recovery. The results indicated that the MIPs were stable in the cycle tests, specifically in more than 20 adsorption–elution cycles with stable SPE recoveries of the target analyte (Figure 4C).

**Figure 5:**
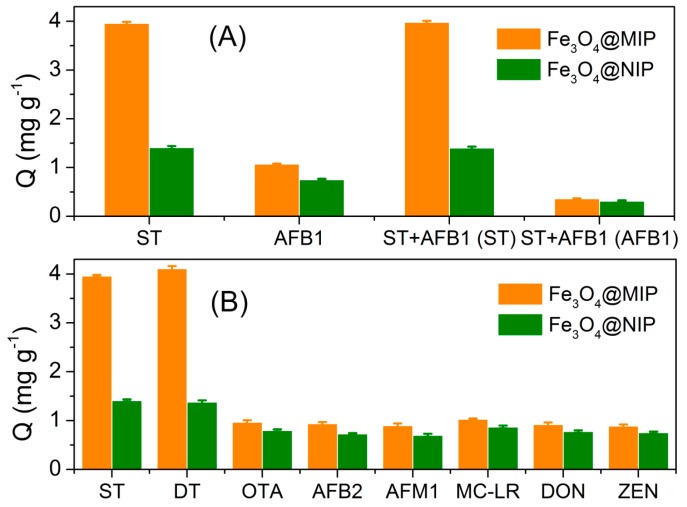
(**A**) Selective and competitive adsorptions of ST and AFB1 by Fe_3_O_4_@MIPs and Fe_3_O_4_@NIPs; and (**B**) specificity of the developed MIP HPLC method for ST determination.

To evaluate the specificity of the developed Fe_3_O_4_@MIPs materials, the imprinting factors are introduced for comparison. The adsorption of the Fe_3_O_4_@MIPs and Fe_3_O_4_@NIPs to both ST and the structural analog, AFB1 (Figure 4D) were carried out at the same experimental conditions. The imprinting factor was defined as the ratio of Q_MIPs_ to Q_NIPs_, which represented adsorption capacity of MIP and NIP, respectively. As shown in Figure 5, the Fe_3_O_4_@MIPs showed a significantly higher adsorption capacity of ST than AFB1, while the Fe_3_O_4_@NIPs did not show such a difference, indicating that the template molecule had a relatively higher affinity for the imprinted polymer than its analog. Moreover, the imprinting factor of ST (2.8) was also much higher than its analog (1.4), further confirming the excellent recognition performance of Fe_3_O_4_@MIPs to ST. Furthermore, co-existing experiments demonstrated similar results as above. Although ST and AFB1 have similar scaffold (xanthene), the differences in their spatial structures and functional groups caused a mismatch in the holes and binding sites, leading to the good selectivity to ST against AFB1. Due to utilizing DT as template for imprinting, the selectivity of DT is comparable to ST. However, in most cases, DT scarcely exist together with ST in grain samples; even during coexistence, the interference could be effectively reduced or totally eliminated via the extraction procedures [22]. Besides, owing to the well-constructed imprinting polymers, the Fe_3_O_4_@MIPs showed insignificant response to the interferents, such as OTA, AFB2, AFM1, MC-LR, DON, and ZEN, compared with that of target ST.

The proposed fluorescent MIPs method gave a linear range of 0.05–5.0 mg·L*^−^*^1^ with a detection limit (3 s) of 9.1 μg·L*^−^*^1^ for the detection of ST. The precision (relative standard deviation (RSD)) for eleven replicate detections of 0.5 mg·L*^−^*^1^ ST was 2.1%. These results indicate that the Fe_3_O_4_@MIPs can be used for the sensitive and selective SPE and determination of ST in complex samples. Compared with the previously-reported methods for ST determination in terms of sensitivity and linear range, the developed MIP method showed comparable performance (Table 2).

### 3.3. Real Sample Analysis

**Figure 6:**
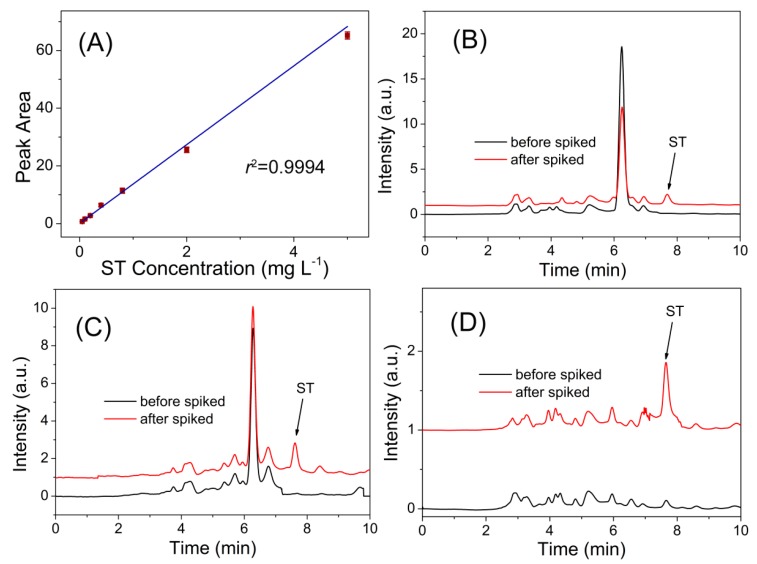
(**A**) The linear response of the developed MIP-HPLC method to ST. (**B**–**D**) Chromatograms of blank sample (black line) and spiked with 50.0 ug·kg^−1^ ST (red line) for analyses of: wheat (**B**); rice (**C**); and millet (**D**).

To demonstrate the applicability of the developed magnetic MIP-HPLC method for real sample analysis, it was applied for the selective isolation and enrichment of ST in food samples via spiked recovery experiments. As can be seen in the chromatograms of the cereal samples before and after being spiked with ST at 50 μg·kg*^−^*^1^, ST appeared at 7.86 min after being spiked, and other irrelevant compounds in the sample showed insignificant interference to the measurement (Figure 6). As shown in Table 3, the obtained recoveries of the spiked samples were in the range of 85.2–88.1% for wheat, 80.6–88.7% for rice, and 82.9–88.6% for millet, with relative standard deviation (RSD) less than 5.6%.

## 4. Conclusions

In this study, MIPs were synthesized onto Fe_3_O_4_@SiO_2_ magnetic particles with a uniform core–shell structure by surface imprinting and nanotechnology. The Fe_3_O_4_@MIPs showed remarkable specificity target ST along with sensitive response. The successful selective separation and enrichment of ST in food samples indicated that the Fe_3_O_4_@MIPs was ideal solid-phase extraction material and had the potential of applying to detect the illegal addition of ST in food.

## Figures and Tables

**Table 1:** Evaluation of adsorption isotherm by the Langmuir and Freundlich models, and data for pseudo-first-order and pseudo-second-order rate constant, experimental *q_e_* values, intra-particle diffusion rate constant, and Elovich parameters.

**Freundlich Model**			**Langmuir Model**		
*K_F_*	*n*	*R*^2^	*Q_m_*	*R_l_*	*R*^2^
3.12	1.67	0.9486	0.92	0.85	0.9948
**Pseudo-First-Order Kinetics**			**Pseudo-Second-Order Kinetics**		
*K*_1_ (min^−1^)	*q_e_* (*cal*) (mg·g^−1^)	*R*^2^	*K*_2_ (min^−1^)	*q_e_* (*cal*) (mg·g^−1^)	*R*^2^
0.045	0.53	0.9726	0.019	0.91	0.9923
**Intra-Particle Diffusion**			**Elovich**		
*k_int_* (mg·g^−1^·min^−1/2^)	*C* (mg·g^−1^)	*R*^2^	*α* (mg·g^−1^·min^−1^)	*β* (g·mg^−1^)	*R*^2^
0.2987	1.3782	0.8832	0.98	0.88	0.9529

**Table 2:** Comparison of the developed magnetic MIP method with the previously-reported methods for ST determination.

Method	Detection Limit (μg·L^−1^)	Linear Range (μg·L^−1^)	Reference
ELISA	0.36	NA	[6]
GC-MS	3	10–150	[3]
LC-MS	3	NA	[5]
LC-MS	3	NA	[7]
HPLC	9	50–5000	This Work
Fluorescence	19	50–2000	[22]
Fluorescence	13	20–1000	[24]

GC-MS: gas chromatography-mass spectrometry; LC-MS: liquid chromatography-mass spectrometry; HPLC: high-performance liquid chromatography; NA: Not applicable.

**Table 3:** Application of the developed MIP-HPLC method for the determination of ST in real samples.

Samples	Spiked Amount (μg·kg^−1^)	Determined Amount (μg·kg^−1^, Mean ± SD)	Recovery (%)	RSD (%)
Wheat	50	44.1 ± 0.9	88.1 ± 1.7	1.1
	100	85.2 ± 4.8	85.2 ± 4.8	5.6
	200	174.4 ± 0.7	87.2 ± 0.4	0.4
Rice	50	43.6 ± 1.9	87.2 ± 3.7	4.4
	100	88.7 ± 1.1	88.7 ± 1.1	1.2
	200	161.2 ± 1.4	80.6 ± 0.7	0.9
Millet	50	41.5 ± 1.7	82.9 ± 3.5	4.1
	100	88.6 ± 1.4	88.6 ± 1.4	1.6
	200	170.8 ± 9.3	85.4 ± 4.6	5.4

SD: standard deviation; RSD: relative standard deviation.
